# Photoactive hydrogels for pre-concentration, labelling, and controlled release of proteins†

**DOI:** 10.1039/d3an00811h

**Published:** 2023-07-26

**Authors:** Leanne Kellermann, Ruchi Gupta

**Affiliations:** a School of Chemistry, University of Birmingham Birmingham, B15 2TT United Kingdom r.gupta.3@bham.ac.uk

## Abstract

We report a novel hydrogel for pre-concentration, fluorescent labelling, and light-triggered release of proteins for detection of low abundance biomarkers. The hydrogel was a co-polymer of acrylamide/bisacrylamide and methacrylamide attached to fluorescein isothiocyanate *via* a light cleavable bond and a poly(ethylene glycol) spacer arm of molecular weight of 3400 g mol^−1^. Unlike previous work, proteins were captured by an irreversible chemical reaction rather than by non-covalent affinity binding or physical entrapment. Because the protein-reactive group was attached to fluorescein, which in turn was coupled to the hydrogel by a photocleavable bond, on release the protein was labelled with fluorescein. Our hydrogel offered a pre-concentration factor of up to 236 for a model protein, streptavidin. Each protein molecule was labelled with 85 fluorescein molecules, and 50% of the proteins in the hydrogel were released after UV exposure for ∼100 s. The proteins released from the hydrogel were captured in biotinylated microtitre plates and detected by fluorescence, allowing measurement of at least 0.01 ppm (or ∼166 pM) of protein in sample solutions. The reported hydrogel is promising for detection of low abundance proteins while being less laborious than enzyme-linked immunosorbent assay and less affected by changes in environmental conditions than label-free biosensors.

## Abstract 2

We report a novel hydrogel for pre-concentration, fluorescent labelling, and light-triggered release of proteins for detection of low abundance biomarkers.

## Graphical abstract

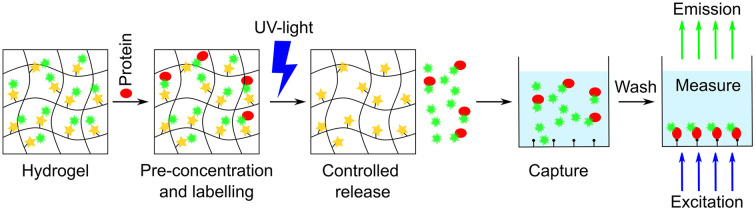

## 1.Introduction

Much of the work on entrapment and release of molecules from hydrogels has been concerned with control of drug delivery.^1–5^ In contrast, less work has been reported on the use of functional hydrogels for analytical purposes. Measurement of low abundance proteins is key for enabling early detection of diseases.^6^ A widely used method for the measurement of low abundance proteins is enzyme-linked immunosorbent assay (ELISA).^7^ ELISA uses two antibodies where a capture antibody allows a target protein to be pulled out of samples and a detection antibody is labelled with an enzyme. A substrate is then added, which is acted upon by the enzyme to produce a fluorescent or coloured product. The fluorescence intensity or absorption is proportional to the concentration of the target protein. Furthermore, as each enzyme can act upon multiple substrate molecules, the signal is amplified, allowing measurement of low abundance proteins. ELISA, however, requires multiple adding and washing steps, and hence is laborious. The technique can be automated, but robotic handling systems are large and expensive. To address this challenge, ELISA has been implemented on microfluidic paper-based analytical devices by controlling the flow rate and hence arrival times of reagents to the detection zone.^8^ The flow control requires patterning of paper and multiple layers which must be precisely aligned. Alternatively, label-free biosensors have been used to measure low abundance proteins. Unlike ELISA, label-free biosensors^9^ require only capture antibodies and rely on changes in refractive index,^10–12^ impedance^13,14^ or mass^15,16^ to determine the concentration of target proteins. However, label-free biosensors are prone to errors because of changes in environmental conditions such as temperature.^17^ To measure low abundance proteins using absorption or fluorescence, which are less sensitive but require simpler instrumentation and are more user-friendly than ELISA, proteins have been pre-concentrated using beads^18^ and electrophoresis.^19–21^ After pre-concentration, proteins are recovered, labelled with dyes or fluorophores, captured, and then detected. This implies that a series of disparate steps are currently needed, much like ELISA.

Previous work on the use of hydrogels for analysis has used non-covalent interactions with immobilised dyes,^22^ charged groups,^23^ cyclodextrins,^24^ nanoparticles^25^ and molecular imprinting^26^ to concentrate proteins in hydrogels. Release of the captured proteins has been by changing the swelling of the hydrogel by pH,^3^ temperature change^1,2^ or by degradation of the hydrogel using light^27–29^ or chemically.^30,31^ For example, Douglas *et al.*^22^ used *N*-isopropylacrylamide (NIPAm) – acrylic acid (AAc) copolymer microparticles modified by a dye (Acid black 48) covalently bound to the hydrogel. The dye acted as an affinity reagent to non-covalently bind proteins. Although this approach preconcentrated proteins from urine, it did not label them on release, the microparticles were extracted from the sample by centrifugation and the release was performed using an elution buffer which diluted the captured proteins. Detection of the captured and released proteins was by western blotting. The use of the microparticles for detection of proteins characteristic of Lyme disease did, however, improve the limit of detection by a factor of 10, from 0.07 ppm without microparticles to 0.007 ppm using microparticles.

**Fig. 1:**
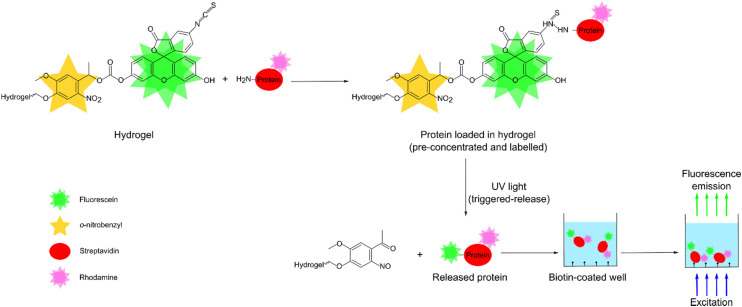
Schematic showing pre-concentration, labelling and release of an exemplar protein, rhodamine-streptavidin, followed by fluorescence detection in a biotin coated microtiter plate.

Herein, we report a novel hydrogel for pre-concentration, fluorescent labelling, and light-triggered release of proteins. The pre-concentration was achieved by covalent capture of proteins in hydrogels having much smaller volumes than sample solutions. As shown in Fig. 1, the covalent capture of proteins was because of the reaction between primary amines in proteins with isothiocyanate group in fluorescein isothiocyanate (FITC)^32,33^ present in the hydrogels. As FITC is fluorescent, proteins were pre-concentrated and labelled in a single step. Furthermore, as shown in Fig. 1, the FITC was attached to the hydrogel backbone *via* a photolabile group, *o*-nitrobenzyl,^34–40^ which exhibits controllable photoreactions with tunable absorption for wavelengths >300 nm.^38,40^ Thus, the release of the fluorogenically labelled proteins was triggered through the irradiation of the hydrogels with UV light (365 nm). As shown in Fig. 1, the released proteins were selectively captured and then measured using fluorescence detection.

To demonstrate the feasibility of our designed hydrogel for pre-concentration, labelling, and controlled release, streptavidin was used as an exemplar protein. The streptavidin used in this work was tagged with rhodamine so that fluorescence of rhodamine could be used to determine the protein concentration before and after incubation with our hydrogels. This in turn provided the pre-concentration factor. The designed hydrogels offered a pre-concentration factor of up to 236. Equally, the rhodamine label was used to study the light-triggered release kinetics of streptavidin from the hydrogels. We showed that 50% of streptavidin was released from our hydrogels in ∼100 s. Finally, we showed that released streptavidin was labelled with 85 fluorescein molecules per molecule of the protein. The designed hydrogel when combined with capture of released streptavidin using biotin and fluorescence detection, allowed detection of at least 0.01 ppm (or ∼166 pM) of the protein.

## 2.Experimental

### 2.1 Materials and equipment

Allyltrichlorosilane (95%), acrylamide/bis-acrylamide (40% solution, 29 : 1), ammonium persulfate (≥98%), ethanol (≥98%, Supelco), fluorescein, fluorescein isothiocyanate isomer I (FITC) (≥90%), poly(ethylene glycol) (PEG) bis(amine) (average *M*_n_ 400 and 3400), 4-(4-(1-hydroxyethyl)-2-methoxy-5-nitrophenoxy)butanoic acid (NVOC), (1-[bis(dimethylamino)methylene]-1*H*-1,2,3-triazolo[4,5-*b*]pyridinium 3-oxide hexafluorophosphate (HATU) (97%), *N*,*N*-diisopropylethylamine (DIPEA) (98%), triethylamine (TEA) (99%), methacryloyl chloride (97%), phosphate-buffered saline (PBS, 10 mM, pH 7.4), phosgene solution (15 wt% in toluene), silica gel (high-purity grade, pore size 60 Å), sodium sulphate, methanol, ethyl acetate, hexane, DMSO-d_6_ (99.9%, MagniSolv), CDCl_3_ (99.9%, MagniSolv), and acetone-d_6_ (99.9%, MagniSolv) were bought from Merck. Decon 90 was bought from Fisher Scientific. The remaining solvents were bought from Merck and prepared before use. Tetrahydrofuran (THF) and toluene were distilled under nitrogen from sodium using benzophenone as indicator. Dichloromethane (DCM), dimethylformamide (DMF) and acetonitrile were distilled under nitrogen from calcium hydride. Streptavidin tagged with rhodamine (S6366) was bought from Thermo Fisher.

Thin layer chromatography was performed on Macherey Nagel aluminum TLC-plates, pre-coated with 0.20 mm silica gel 60 and fluorescent indicator UV 254. Visualization was performed with iodine on silica, followed by heating and UV light of wavelength 254 nm. Slide-A-Lyzer Dialysis Cassettes (MWCO 2K, Thermo Fisher) were used for purification of PEG 3400 monomers. Pierce™ Biotin Coated Plates (Clear, 8-Well Strip, Thermo Scientific) were used for protein release studies. Microscope glass slides (1 mm thick low iron standard) were purchased from VWR (Leicestershire, UK).

^1^H and ^13^C NMR spectra were obtained at 25 °C and recorded on a 300 MHz Varian VNMRS, 400 MHz Varian Unity Inova or 600 MHz Varian Unity Inova. Chemical shifts (*δ*) are reported in parts per million (ppm), and splitting patterns are designated as s (singlet), d (doublet), dd (doublet of doublets), t (triplet), q (quartet), m (multiplet) and br s (broad singlet) and coupling constants (*J*) are expressed in Hertz (Hz). ^1^H and ^13^C NMR spectra are referenced to the residual solvent signal: DMSO-d_6_ (2.50 or 39.52 ppm) and CDCl_3_ (7.26 or 77.16 ppm).

Irradiation at 365 nm was carried out using an in-house constructed source based on a UV LED (Nichia NVSU233A-U365, RS Components) with a radiant flux of 1030 mW at 1A forward current expanded to 25 mm diameter, giving a flux density of ∼210 mW cm^−2^. The LED and DC-DC converter components driving the LED were mounted on a 60 × 60 mm^2^ aluminum–cored PCB, which in turn was mounted to a 60 × 60 mm^2^ heat sink and fan (Thermo Electric Devices TDEX6015/TH12G, RS Components) using thermally conductive paste. The LED driver and fan were powered by a 12 V 1A DC mains adaptor.

UV–Vis spectroscopy measurements were carried out on a Jenway 6715 UV–Vis spectrometer at 25 °C. High-performance liquid chromatography (HPLC) was performed using Agilent 1260 Infinity coupled with a Vanquish™ multi wavelength absorbance detector and in a C18 column (1.8 μm particle size, 3 mm internal diameter and 50 mm long). A CLARIOstar Plus microplate reader was used to measure the fluorescence intensity of excited samples (fluorescein, *λ*_excitation_ = 472 nm, emission measured = 500–620 nm; rhodamine *λ*_excitation_ = 540 nm, emission measured = 570–670 nm).

### 2.2 Monomer synthesis and purification

**Fig. 2:**
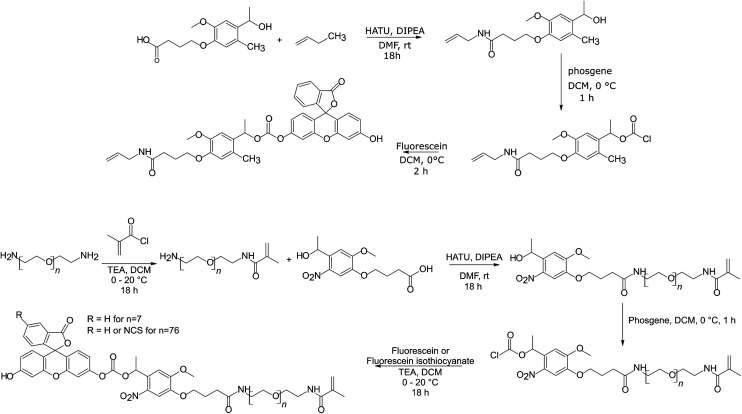
Reaction schemes for the synthesis of monomers.

The reaction schemes used to synthesize F-NVOC-allylamide, F-NVOC-PEG_400_-methacrylamide, F-NVOC_3400_-methacrylamide, and FITC-NVOC_3400_-methacrylamide are shown in Fig. 2. The reaction schemes were adapted from the procedure reported by Landfester and Klinger^40^ and our previous work.^41,42^ The details of reaction and purification conditions of monomers along with characteristic shifts in their NMR spectra are provided in the ESI.†

### 2.3 Preparation of hydrogel films

Glass microscope slides were cut into 25.4 ± 0.5 mm^2^ and cleaned in Decon 90, water, and ethanol for 30 min each in an ultrasonic bath. The glass squares were immersed in 0.5% v : v allyltrichlorosilane in toluene for 30 min, washed in toluene, and dried before use. 1 mL solutions containing either F-NVOC-allylamide dissolved in 250 μL DMSO or (F or FITC)-NVOC-PEG_(400 or 3400)_-methacrylamide dissolved in 250 μL of deionized water, 40% w : v acrylamide/bis-acrylamide (volume calculated to create a 10 wt% gel), 1.25 μL of TEMED, and 12.5 μL of 10% w : v APS were prepared in N_2_-degassed water. The concentration of the NVOC containing monomer was 5, 10, or 15% of the total molar concentration of the monomers in the precursor solution. The precursor solution was cast between glass squares and plastic covers separated with a spacer of 175 μm thickness (939-837-76, Goodfellow) at room temperature in darkness. After 15 min, plastic covers were removed, and hydrogel films deposited on glass substrates were soaked in PBS overnight to remove any residual unreacted monomers.

### 2.3 Characterization of monomers and hydrogel films

The UV-Vis absorption spectra of monomers were measured at different concentrations to determine their molar extinction coefficient. Monomer stock solutions were irradiated with 365 nm light and samples were taken at fixed time intervals for subsequent analysis by UV-Vis spectroscopy and HPLC. For HPLC, 1 mg ml^−1^ monomer stock solutions were prepared in acetonitrile and collected samples solutions were chromatographed using a solvent gradient of acetonitrile/water from 90/10 to 10/90. The chromatograms were recorded at a wavelength of 491 nm. The HPLC data was used to determine the kinetics of light-triggered release of fluorescein from monomers.

The UV-Vis absorption spectra of a hydrogel film (sample 8 in Table S1, ESI†) deposited on a glass slide were measured at different regions to determine area-to-area variability. Equally, absorbance at a wavelength of ∼490 nm of precursor solutions and hydrogel films (see Table S1, ESI† for their composition) were recorded. Using these absorbance values and given the path length of cuvettes containing precursor solutions and thickness of films, we estimated the molar percentage of fluorescein containing monomer incorporated in the hydrogel films. To determine the kinetics of light-triggered release of fluorescein from hydrogel films, the films were exposed to 365 nm light for selected durations, and their UV-Vis spectra were measured after each irradiation time interval.

### 2.4 Protein studies

Protein studies were carried out using functionalised hydrogels made by co-polymerizing FITC-NVOC-PEG_3400_-methacrylamide with acrylamide/bisacrylamide. The percentage molar fraction of FITC-NVOC-PEG_3400_-methacrylamide was 10% and total concentration of monomers in the precursor solution was 10% w : v. Hydrogel films made by polymerizing 10% w : v acrylamide/bisacrylamide served as negative controls. UV-Vis and fluorescence emission spectra of different concentration of rhodamine–streptavidin (RS) in PBS were measured. Hydrogel films of acrylamide/bisacrylamide without and with FITC-NVOC-PEG_3400_-methacrylamide were submerged in a 10 mL stock solution of 0.01 or 0.1 ppm RS in PBS overnight. Fluorescence emission spectra of PBS before and after incubation with each type of hydrogel films were measured. The data was used to determine the factor by which proteins were pre-concentrated.

The hydrogel films were washed in fresh PBS at room temperature in dark conditions overnight to remove any unbound RS. Subsequently, the films were immersed in 3 mL PBS and exposed to UV-light irradiation in short time bursts of between 1 and 10 min. After each irradiation, the hydrogel was left in PBS in darkness for 30 min to allow the released RS to diffuse out of hydrogel films, and then the PBS was decanted from the hydrogel. The hydrogel films were then immersed in 3 mL of fresh PBS and the above process was repeated. The light-triggered release kinetics of RS was studied by monitoring the fluorescence emission corresponding to rhodamine with irradiation times. All measurements were performed in triplicate. To verify that the released RS is labelled with both rhodamine and fluorescein, 200 μl of the PBS solution collected after UV-light exposure was pipetted in a biotin coated well of a microtiter plate and incubated for 30 min. Subsequently, the well was washed with 200 μL of PBS and the fluorescence emission spectra were measured at excitation wavelengths of 470 and 540 nm corresponding to fluorescein and rhodamine, respectively.

## 3.Results and discussion

### 3.1 Monomers

**Fig. 3:**
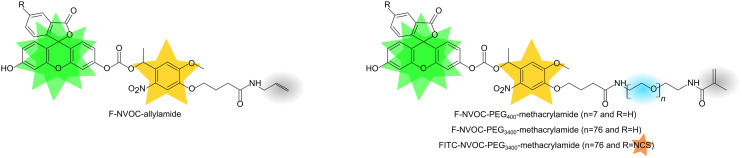
Chemical structures of monomers (orange, green, and yellow stars are groups for protein capture, fluorescent labelling, and light triggered release, respectively, blue circle is PEG, resulting in water soluble monomers, and grey circle is allylamide or methacrylamide, allowing monomers to be co-polymerised with acrylamide/bisacrylamide to obtain hydrogels).

The chemical structures of the synthesized monomers are provided in Fig. 3. Fig. S1, ESI† shows that the peak absorbance wavelength for F-NVOC-allylamide dissolved in DMSO was observed at 519 nm with a shoulder at ∼490 nm, suggesting aggregation. The aggregation was further supported by the observation that F-NVOC-allylamide solution in DMSO was largely non-fluorescent. In contrast, as shown in Fig. S2 and S3, ESI† F-NVOC-PEG_400_-methacrylamide and F-NVOC-PEG_3400_-methacrylamide monomers were water soluble and absorbed at wavelengths of ∼490 nm and ∼365 nm, which was attributed to fluorescein and NVOC groups.

**Fig. 4:**
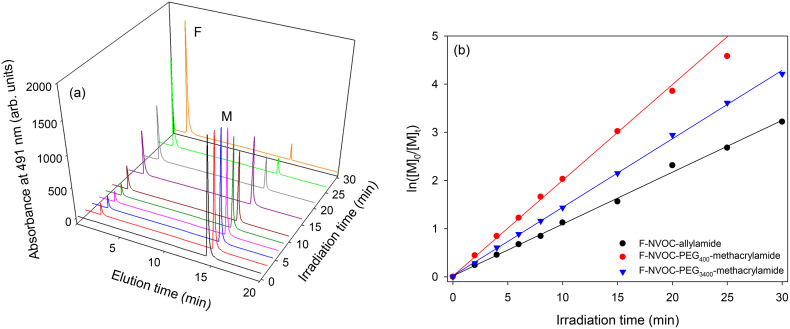
(a) HPLC waterfall plot of F-NVOC-PEG_3400_-methacrylamide showing increasing release of fluorescein (peak F) and decrease of starting reagent (peak M) as a function of irradiation time, and (b) rates of the light induced release of fluorescein from monomers with increasing irradiation time, where [M]_*t*_ is the concentration of monomer as a function of irradiation time and [M]_*0*_ is the starting monomer concentration.

Photolysis occurs when the irradiation wavelength overlaps with the absorption band of the photolabile group,^43–45^ in this case, NVOC. Thus, the monomers solutions were irradiated to 365 nm UV-light. Typical UV-Vis spectra of the monomers for different irradiation times (see Fig. S4, ESI†) showed that the peak attributed to the NVOC group was red shifted, indicating that nitrosobenzaldehyde was produced and hence the photoreactions were successful. The absorption at ∼490 nm remained unchanged with irradiation times because both released fluorescein and fluorescein bound to the monomer remained in the same solution. Thus, released fluorescein and the monomer were separated using HPLC with the resulting chromatograms provided in Fig. 4(a) and Fig. S5 and S6, ESI.† HPLC measurements were carried out in acetonitrile after a range of irradiation times. The kinetics of light-triggered release of fluorescein was investigated by monitoring the rate of disappearance of the monomers ([M]_*t*_). Plots of ln[M]_0_/[M]_*t*_*versus* irradiation time (see Fig. 4(b)) show excellent linearity, indicating the expected first order kinetics. Furthermore, the rate plots provided in Fig. 4(b) show that kinetics of light-triggered release of fluorescein from PEG-methacrylamide monomers was faster than the allylamide monomer.

### 3.2 Hydrogel films

**Fig. 5:**
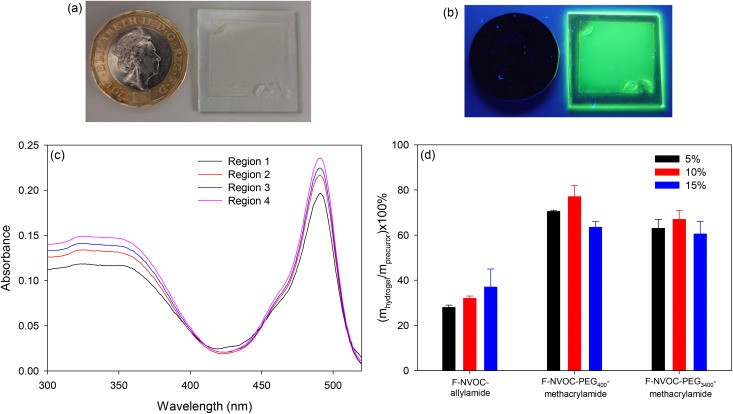
Images of a hydrogel film on glass under (a) white, and (b) 365 nm light with a £1 coin as a size reference (distance across flats 22.5 mm), (c) UV-Vis spectra of four different regions of the hydrogel film, and (d) graph showing monomer incorporation factors, which is the ratio of the molar concentrations of the NVOC containing monomers in hydrogels (*m*_hydrogel_) and precursor (*m*_precursor_) solutions.

Hydrogels were prepared by free radical co-polymerisation of acrylamide/bisacrylamide with different monomers. The compositions of hydrogels studied in this work are summarized in Table S1, ESI.† The hydrogels were cast on a glass slide for support and the ability to place the slide inside a UV-Vis spectrophotometer. Fig. 5(a) shows a hydrogel film on a glass slide under white light with a £1 coin as a size reference, Fig. 5(b) is the same slide under 365 nm illumination showing the emission of the fluorescein incorporated in the hydrogel. Fig. 5(c) gives the UV-visible absorption spectra of different regions of that slide, showing that the thickness of the film is not quite uniform. These spectra were obtained by changing the position of the slide relative to the beam of the spectrophotometer. Fig. 5(d) gives the monomer incorporation factors for the different types and molar ratios of the NVOC containing monomers. The incorporation factor was defined as the ratio of the molar concentrations of the NVOC containing monomers in hydrogels (*m*_hydrogel_) and precursor solutions (*m*_precursor_). The *m*_hydrogel_ and *m*_precursor_ were estimated based on the absorbance of the hydrogel and precursor solutions at ∼490 nm and using the molar extinction coefficient of fluorescein (Fig. S7, ESI†). As shown in Fig. 5(d), the PEG-based methacrylamide monomers exhibited increased monomer incorporation factors when compared to the allylamide monomer. This was attributed to an increase in monomer reactivity (methacrylamide > allylamide)^46^ and the water solubility of the PEG-based monomers. There was a slight decrease in monomer incorporation with increasing PEG molecular weight.

**Fig. 6:**
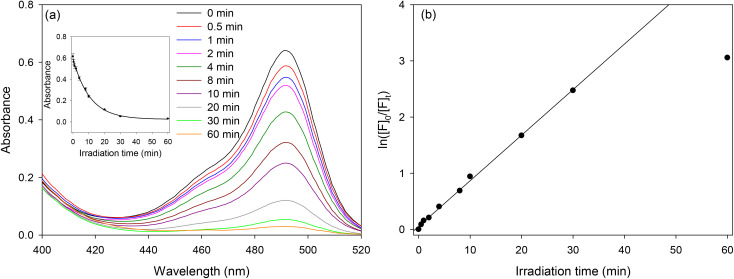
(a) UV-Vis spectra of a hydrogel film (co-polymer of acrylamide/bisacrylamide and F-NVOC-PEG_3400_-methacrylamide) with increasing irradiation times, and (b) first-order reaction rate plot of the release of fluorescein from this hydrogel.

The photoinduced release of fluorescein from hydrogels was investigated next. Fig. 6(a) shows the decrease of the absorption band of fluorescein in the hydrogel film of slide 8 (Table S1, ESI†) as the irradiation time increases. Fig. 6(b) shows the first-order reaction rate plot of the decrease in fluorescein concentration in this hydrogel, indicating that the release is first-order up to 30 min of irradiation. Similar results were obtained for the other prepared hydrogels, showing first-order release kinetics up to 30 min of irradiation. This suggests that fluorescein, once released, can rapidly diffuse out of the hydrogel, but the non-linearity after ∼30 min irradiation suggests that there might have been a small amount of irreversibly bound fluorescein remaining in the hydrogel.

In summary, F-NVOC-PEG_3400_-methacrylamide could be prepared in high yield (59% compared to only 35% for F-NVOC-PEG_400_-methacrylamide) and did not require purification by flash chromatography. Furthermore, the kinetics of the release of fluorescein in solutions of F-NVOC-PEG_3400_-methacrylamide was the fastest. Finally, the percentage of monomer incorporated in the hydrogel with respect to the precursor solution was higher for F-NVOC-PEG_3400_-methacrylamide than F-NVOC-allylamide (65% and ∼32%, respectively). Considering these benefits offered by F-NVOC-PEG_3400_-methacrylamide, the protein studies were carried out using a derivative of this monomer, FITC-NVOC-PEG_3400_-methacrylamide, with an isothiocyanate group that could react with the primary amines in proteins.

### 3.3 Protein studies

Rhodamine–streptavidin (RS) was selected as an exemplar protein to study pre-concentration, labelling and release of proteins followed by their quanttation using fluorescence in biotin coated microtiter plates. The streptavidin used in this work was tagged with rhodamine so that fluorescence of rhodamine could be used to determine the protein concentration before and after incubation with hydrogels. This in turn provided the pre-concentration factor. Streptavidin was chosen because of its very strong and selective binding to biotin, which made it easy to capture in biotinylated microtiter plates after being released from hydrogels.

#### 3.3.1 Protein capture and pre-concentration

**Fig. 7:**
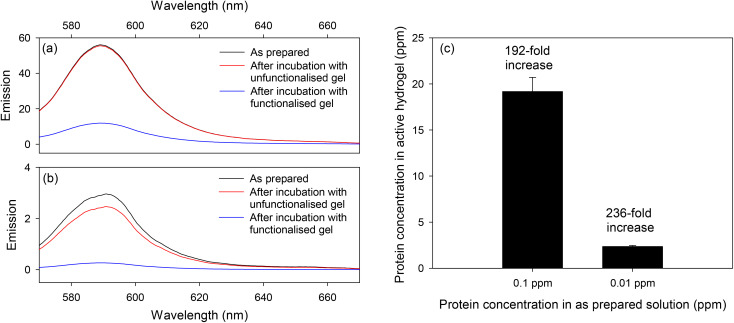
Emission spectra of (a) 0.1 and (b) 0.01 ppm RS solutions before and after overnight incubation with unfunctionalized and functionalized hydrogels, and (c) RS concentrations in solution and hydrogel.

The functionalized hydrogels, which were prepared by co-polymerizing acrylamide/bisacrylamide and FITC-NVOC-PEG_3400_-methacrylamide monomers, were capable of protein capture because the isothiocyanate groups reacted with the terminal amine and primary amines in proteins.^47^ This was confirmed by submerging the formed hydrogels in 10 mL of 0.10 and 0.01 ppm (1.66 and 0.166 nM, respectively) RS solutions in PBS. As shown in Fig. 7, a majority of RS was lost from the solution after overnight incubation with a functionalized hydrogel. In contrast, control experiments with unfunctionalized polyacrylamide hydrogels resulted in no significant loss of RS from solutions. The negligible reduction in fluorescence from the stock solutions exposed to the unfunctionalized hydrogels shows that the RS conjugate does not bind significantly in the absence of the FITC moiety (Fig. 7).

**Fig. 8:**
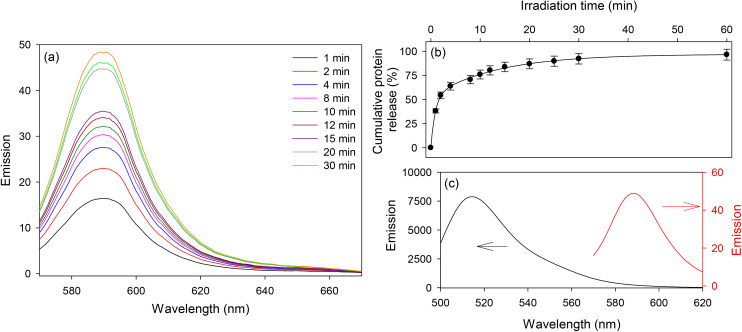
(a) Emission spectra of RS (excitation wavelength of 540 nm) released when hydrogels were irradiated with UV-light, (b) cumulative release of protein as a function of irradiation time, and (c) emission spectra of released after incubation with biotin coated microtiter plates and buffer wash (excitation wavelengths of 470 and 540 nm).

Six functionalized hydrogels were prepared; three were incubated with 0.1 ppm RS solutions and the remaining three with 0.01 ppm RS solutions. As summarized in Table 1, the percentage of RS loaded in functionalized hydrogels was 77 ± 8% and 94 ± 6% for 0.1 and 0.01 ppm protein solutions, respectively. This is significant when compared to the light-responsive hydrogels prepared by Klinger and Landfester,^40^ where proteins were trapped inside hydrogels as a result of decreased pore size arising from electrostatic interactions, where a protein loading of 53.2 ± 7.4% was reported. Subsequently, the concentration of RS in functionalized hydrogels was estimated and the results are provided in Table 1. The ratio of concentrations of RS in hydrogels and stock solutions provided the pre-concentration factors, which as shown in Fig. 8(c), were estimated to be 192 and 236 for 0.1 and 0.01 ppm RS solutions, respectively. The slightly lower pre-concentration factor at higher protein concentration is though to be a result of saturation of the reactive groups in the hydrogel.

**Table 1:** A summary of RS loading in hydrogels made by co-polymerizing FITC-NVOC-PEG_3400_-methacrylamide with acrylamide/bisacrylamide (molar ratio of FITC-NVOC-PEG_3400_-methacrylamide was 10%, volume of RS stock solution was 10 mL, volume of hydrogel was estimated to be 0.04 mL, and ‡: determined by change in fluorescence emission)

RS concentration before incubation with functionalized hydrogel (ppm)	RS concentration after overnight incubation with functionalized hydrogel (ppm)‡	% RS loaded in functionalized hydrogel	Amount of RS in functionalised hydrogel (μg)	RS concentration in functionalised hydrogel (ppm)
0.10	0.0176	82%	0.824	20.6
	0.0205	80%	0.795	19.9
	0.0318	68%	0.682	17.1
0.01	0.0003	97%	0.097	2.4
	0.0012	88%	0.088	2.2
	0.0002	98%	0.098	2.5

#### 3.3.2 Protein release and detection

Having demonstrated the loading of a model protein into the prepared hydrogels, the photolytic release of protein from the hydrogels was investigated. The loaded hydrogels were exposed to UV in short bursts of between 1 and 10 min while submerged in 3 mL PBS, kept in PBS for 30 min to allow for the released RS to diffuse out of the hydrogel, and then the PBS was collected. This process was repeated until no additional RS could be detected in the supernatant PBS.

The fluorescence emission spectra of the PBS collected after each irradiation are shown in Fig. 8(a). The resulting cumulative release of RS as a function of irradiation time is shown in Fig. 8(b). The experimental data shown in Fig. 8(b) when fitted to a single exponential rise did not give a good fit to the data (*r* = 0.966), but a double exponential rise to a maximum gave a good fit with a correlation coefficient of 0.9995, indicating that two processes take place during the photolytic release of captured RS. The first fast process accounts for about 54% of the RS released with a time constant of 1.124 min^−1^, while the second slower process accounts for the remaining 46% of the released RS with a time constant of 0.075 min^−1^. From this we can show that 50% of the RS was released in ∼1.68 minutes, or ∼100 s. The origin of these two processes is currently unclear but may be a result of slow diffusion of released RS from the deeper layers of the hydrogel.

One of the PBS solutions containing labelled protein collected from a hydrogel incubated with 0.01 ppm RS and exposed to UV light was dispensed into a biotin coated well of a microtiter plate. The released streptavidin was allowed to bind to biotin for 30 min and then a buffer wash was performed. This process allowed removal of any fluorescein dye, not bound to protein but released when the hydrogel was irradiated with UV-light. The well of the microtiter plate was illuminated with 470 and then 540 nm light. The resulting fluorescence spectra (see Fig. 8(c)) showed emission peaks corresponding to fluorescein and rhodamine, confirming that the released RS was successfully labelled with fluorescein. Fig. S7 and S8, ESI† show that the molar emission coefficient of fluorescein at 514 nm is ∼10 times higher than rhodamine at 590 nm in RS. Based on this and considering the fluorescence emission intensities at 514 and 590 nm in Fig. 8(c), we estimate that the RS was labelled with ∼17 fluorescein molecules per one molecule of rhodamine. As each streptavidin was labelled with 5 rhodamine molecules, we estimate that 85 molecules of fluorescein were attached to each streptavidin. One possible explanation of this observation is that the FITC was separated from the backbone of the functionalized hydrogels by PEG_3400_ spacer arm, making it easier for many FITC molecules to react with each RS molecule loaded in the hydrogels. The labelling of RS with large numbers of fluoresceins is beneficial for measurement of low abundance proteins.

## Conclusions

This work reports a hydrogel for pre-concentration, labelling, and controlled release of proteins for their subsequent detection by fluorescence. The hydrogel was formed by co-polymerizing acrylamide/bisacrylamide with designed monomers. The designed monomers comprised of either fluorescein (F) or fluorescein isothiocyanate (FITC) attached to a polymerizable group *via* a light cleavable bond achieved using *o*-nitrobenzyl (NVOC). The polymerizable group was either allylamide or methacrylamide. A poly(ethylene glycol) (PEG) spacer arm may or may not be present between the NVOC and the polymerizable group. We showed that the incorporation of methacrylamide monomer with 3400 g mol^−1^ molecular weight PEG spacer arm (F-NVOC-PEG_3400_-methacrylamide) in hydrogels was ∼65%, which was double that of the allylamide monomer without PEG. The other benefits offered by the F-NVOC-PEG_3400_-methacrylamide were water solubility, ease of preparation with high yield, and the fastest light-triggered release kinetics.

By replacing fluorescein with FITC in the monomers and subsequently the hydrogels, a model protein was captured by reaction of amines on the protein with the isothiocyanate group. The designed hydrogels offered a pre-concentration factor of 192 and 236 for 0.1 and 0.01 ppm of an exemplar protein, streptavidin. Once pre-concentrated, the proteins were released by UV irradiation, leaving a free protein with a fluorescein label. We showed that 50% of streptavidin was released from our hydrogels in ∼100 s and was labelled with 85 fluorescein molecules per one molecule of the protein. The labelling of proteins with large numbers of fluorophores can allow measurement of proteins present at low levels. Finally, the designed hydrogel when combined with capture of released streptavidin using biotin and fluorescence detection, allowed detection of at least 0.01 ppm or ∼166 pM of the protein.

The hydrogels developed in this work are promising for protein preconcentration, labelling and on-demand release. The developed hydrogel when used in combination with selective capture of labelled proteins and fluorescence detection offer potential for measurement of low abundance proteins in clinical samples, enabling early detection of diseases.

## Author contributions

Gupta did conceptualization, funding acquisition, project administration, resources, supervision, writing – review and editing. All other activities were done by both authors. All authors have given approval to the final version of the manuscript.

## Conflicts of interest

There are no conflicts to declare.

## Supplementary Material

AN-148-D3AN00811H-s001
