# Supplementary material for: Photoactive hydrogels for pre-concentration, labelling, and controlled release of proteins
Source: Analyst. 2023 Jul 26;148(17):4127–37. doi: 10.1039/d3an00811h (PMC10440800; doi:10.1039/d3an00811h)
Supplement: AN-148-D3AN00811H-s001 [file AN-148-D3AN00811H-s001.pdf]

Electronic Supplementary Material (ESI) for Analyst.
This journal is © The Royal Society of Chemistry 2023

Supplementary Information

Photoactive hydrogels for pre-concentration, labelling, and controlled release of proteins

*Leanne Kellermann and Ruchi Gupta**

School of Chemistry, University of Birmingham, Birmingham, B15 2TT

F-NVOC-allylamide

NVOC (300 mg, 1.00 mmol, 1 equiv) and anhydrous DMF (5 mL) were added to a nitrogen purged oven dried 25 mL round-bottom flask (RBF). To this solution, allylamine (75 µL, 1.00 mmol, 1 equiv), HATU (381 mg, 1.00 mmol, 1 equiv), and DIPEA (873 µL, 5.00 mmol, 5 equiv) were added. The reaction was left to stir for 18 h. The following day, the solution was diluted with ethyl acetate (50 mL) and transferred to an extraction vessel. The organic phase was washed with DI water (50 mL) three times. Finally, the organic phase was collected, dried over sodium sulphate, filtered, and concentrated in vacuo. The crude was purified via flash chromatography with an eluent of 5% methanol in DCM. This gave the expected product, N-allyl-4-(4-(1-hydroxyethyl)-2-methoxy-5-nitrophenoxy) butanamide (NVOC-allylamide), as a pale-yellow solid (322 mg, 95%).

NVOC-allylamide (135 mg, 0.40 mmol, 1 equiv) was dissolved in dry DCM (5 mL) in a nitrogen purged oven dried 25 mL RBF wrapped in aluminum foil. The mixture was cooled to 0 ºC and phosgene solution (15 wt.% in toluene) (455 mL, 0.60 mmol, 1.5 equiv) was added dropwise over 20 min and subsequently stirred at 0 ºC for an hour. Fluorescein sodium salt (450 mg, 1.20 mmol, 2 equiv) and TEA (83 µL, 0.60 mmol, 1.5 equiv) were dissolved in cold dry DCM (5 mL) in another dry, dark, two-necked RBF. To this fluorescein containing flask, the first reaction mixture was added dropwise under inert conditions for over 30 min. The reaction proceeded for 2 h in an ice bath and then allowed to cool to room temperature overnight. The volume was reduced by flushing the reaction vessel with nitrogen and then purified using flash chromatography (5% to 40% ethyl acetate: hexanes) in a dark fume hood, to give the product as a yellow solid (F-NVOC-allylamide, 225 mg).

^{1}H NMR (400 MHz, DMSO-d$_6$): δ 8.03 (*t*, J = 5.7 Hz, 1H), 7.52 (*s*, 1H), 7.36 (*s*, 1H), 5.79 (*ddt*, J = 17.2, 10.4, 5.3 Hz, 1H), 5.47 (*d*, J = 4.4 Hz, 1H), 5.26 (*qd*, J = 6.2, 4.3 Hz, 1H), 5.11 (*dq*, J = 17.2, 1.8 Hz, 1H), 5.03 (*dq*, J = 10.2, 1.6 Hz, 1H), 4.04 (*t*, J = 6.5 Hz, 2H), 3.90 (*s*, 3H), 3.69 (*tt*, J = 5.5, 1.7 Hz, 2H), 2.69 (*s*, 2H), 2.28 (*t*, J = 7.4 Hz, 2H), 2.00 – 1.92 (*m*, 2H), 1.36 (*d*, J = 6.2 Hz, 3H). ^{13}C NMR (400 MHz, DMSO-d$_6$) δ 171.23, 153.42, 146.27, 138.89, 137.98, 135.50, 114.95, 109.09, 108.36, 68.28, 63.90, 56.05, 40.84, 40.15, 39.94, 39.73, 39.52, 39.31, 39.10, 38.89, 38.24, 31.48, 25.17, 24.67.

F-NVOC-PEG$_{400}$-methacrylamide monomer

First, PEG$_{400}$ bis(amine) (500 mg, 1 equiv) was added to dry DCM (20 mL). The mixture was then cooled to 0 ºC and TEA (15 uL, 0.1 mmol, 1.2 equiv) was added dropwise and the mixture stirred for 1 h under inert conditions. Following this, methacryloyl chloride (12 uL, 0.36 mmol, 0.6 equiv) was added dropwise. The mixture was stirred for 1 and purified by flash chromatography to yield amine-PEG$_{400}$-methacrylamide (M_n = 504 g.mol^{-1}) in 80% yield.

To an ice-cooled suspension of NVOC (150 mg, 0.50 mmol, 1 equiv.), HATU (229 mg, 0.70 mmol, 1.4 equiv) and 183 µL DIPEA (1.05 mmol, 2.1 equiv) in 10 mL of DMF were stirred. Amine-PEG$_{400}$-methacrylamide (281 mg, 0.70 mmol, 1.4 equiv) was dissolved in minimal amount of DMF and added dropwise to the solution of NVOC, HATU, and DIPEA. The reaction mixture was allowed to warm to room temperature and stirred for 18 h. After the reaction was completed, the mixture was treated with water (20 mL) and extracted with ethyl acetate (20 mL x 3). The combined organic layers were washed with brine, dried over anhydrous sodium sulphate, and concentrated to give a crude product, which was purified by column chromatography (100% ethyl acetate). NVOC-PEG$_{400}$-methacrylamide was obtained at 20% yield as a crystallizing oil.

This oil was then redissolved in dry DCM (20 mL) in a dark RBF and stirred at 0 ºC for 30 min under a constant flow of nitrogen. To this RBF, 4 mL of cold phosgene solution (15 wt.% in toluene, 1.5 equiv) was added dropwise over 20 min. The mixture was stirred under dark and inert conditions for 30 min. To another dry dark RBF, FITC (291 mg, 0.75 mmol) and TEA (60 uL, 0.41 mmol) were dissolved in cold dry DCM (5 mL) and stirred for 30 min. To this RBF, NVOC-PEG$_{400}$-methacrylamide solution was added dropwise over 30 min. The mixture was stirred at room temperature for 18 h. The solvent was evaporated through purging with nitrogen in a fume hood and the residue purified with flash chromatography to yield the targeted monomer (F-NVOC-PEG$_{400}$-methacrylamide, 169 mg, 35%).

^{1}H NMR (400 MHz, DMSO-d$_6$) δ 8.01 – 7.88 (*m*), 7.61 (*s*), 7.27 (*t*, J = 5.7 Hz), 7.23 – 7.13 (*m*), 6.58 (*d*, J = 8.9 Hz), 6.37 (s), 5.66 (*t*, J = 1.3 Hz), 5.32 (*p*, J = 1.6 Hz), 4.08 – 4.02 (*m*), 3.93 – 3.87 (*m*), 3.68 (s), 3.36 – 3.30 (*m*), 3.33 (*s*), 3.16 (*t*, J = 6.1 Hz), 3.07 (*q*, J = 5.9 Hz), 2.43 (*q*, J = 7.1 Hz), 2.10 (*d*, J = 1.5 Hz), 1.99 (*t*, J = 6.5 Hz), 1.85 (*t*, J = 1.2 Hz), 1.64 (*d*, J = 0.9 Hz), 1.37 (*dd*, J = 6.2, 2.0 Hz), 1.35 (*s*), 1.24 (*s*), 0.93 (*t*, J = 7.1 Hz). ^{13}C NMR (400 MHz, DMSO-d$_6$) δ 168.14, 156.83, 140.26, 119.67, 82.48, 70.24, 69.29, 63.70, 46.13, 40.47, 40.26, 40.05, 39.84, 39.63, 39.42, 39.22, 18.99.

F-NVOC-PEG$_{3400}$-methacrylamide and FITC-NVOC-PEG$_{3400}$-methacrylamide monomers

Amine-PEG$_{3400}$-methacrylamide (M_n = 3604 g.mol^{-1}) was prepared by reacting PEG$_{3400}$ bis(amine) with methacryloyl chloride by following the same procedure as described above.

Amine-PEG$_{3400}$-methacrylamide (3 g, 1 equiv) was dissolved in 2 mL of DMF. To an ice-cooled suspension of NVOC (399 mg, 1.33 mmol, 1.5 equiv.), HATU (229 mg, 0.70 mmol, 1.4 equiv) and 183 µL DIPEA (1.05 mmol, 2.1 equiv) in 10 mL of DMF was stirred. The amine-PEG$_{3400}$-methacrylamide solution was added dropwise to the solution of NVOC, HATU, and DIPEA. The reaction mixture was allowed to warm to room temperature and stirred for 18 h. The product, NVOC-PEG$_{3400}$-methacrylamide, was purified by immediate precipitation in diethyl ether, redissolving in DCM and final precipitation in diethyl ether. The resulting pellet was dried under vacuum at 40 °C.

This pellet was divided into two and reacted with either fluorescein or FITC using the same procedure described above. The monomers were purified by dialysis and lyophilized, resulting in a sticky, off-white powder (F-NVOC-PEG$_{3400}$-methacrylamide, 680 mg, 59%; FITC-NVOC-PEG$_{3400}$-methacrylamide, 682 mg, 60%).

^{1}H NMR (400 MHz, DMSO-d_6) δ 7.91 (*t*, J = 5.6 Hz), 7.24 (*t*, J = 5.7 Hz), 5.65 (*t*, J = 1.2 Hz), 5.32 (*p*, J = 1.7 Hz), 4.13 – 4.00 (*m*), 3.50 (*d*, J = 141.5 Hz), 3.15 (*t*, J = 6.1 Hz), 3.07 (*q*, J = 6.1 Hz), 2.54 (*s*), 1.84 (*t*, J = 1.2 Hz), 1.63 (*s*), 1.36 (*d*, J = 10.2 Hz). ^{13}C NMR (400 MHz, DMSO-d_6) δ 168.14, 156.83, 140.26, 119.67, 82.48, 70.24, 69.29, 63.70, 46.13, 40.47, 40.26, 40.05, 39.84, 39.63, 39.42, 39.22, 18.99.

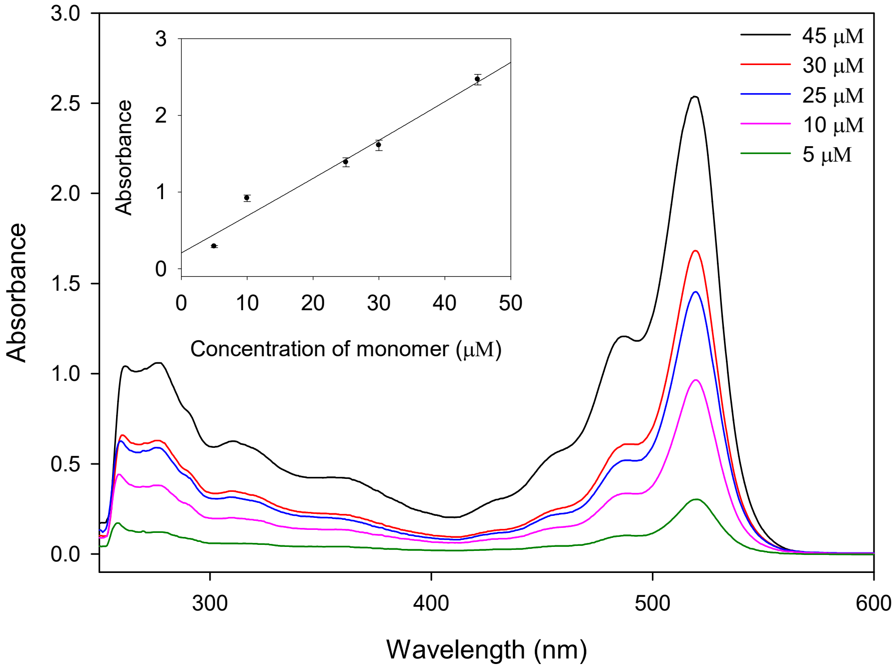

Figure S1: UV-Vis spectra of F-NVOC-allylamide (45, 30, 25, 10 and 5 μM) in DMSO where inset shows average absorbance between ± 5 nm of peak wavelength of 519 nm *versus* the monomer concentration (molar extinction coefficient at 519 nm = 49,600 ± 5,200 $M^{-1}cm^{-1}$)

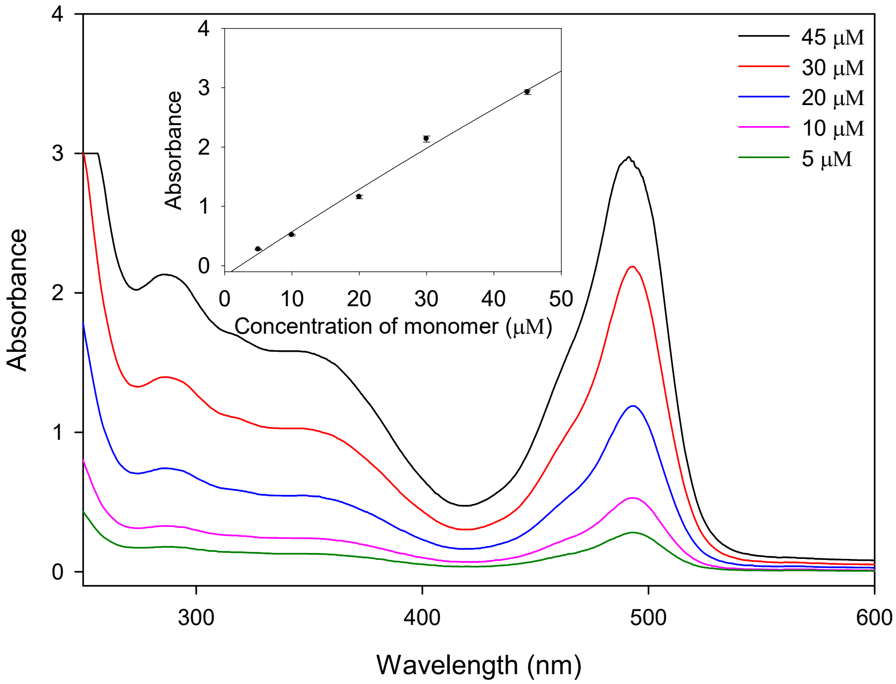

Figure S2: UV-Vis spectra of F-NVOC-PEG_{400}-methacrylamide (45, 30, 20, 10 and 5 μM) in PBS where inset shows average absorbance between ± 5 nm of peak wavelength of 492 nm *versus* the monomer concentration (molar extinction coefficient at 492 nm = 69,100 ± 4,200 $M^{-1}cm^{-1}$)

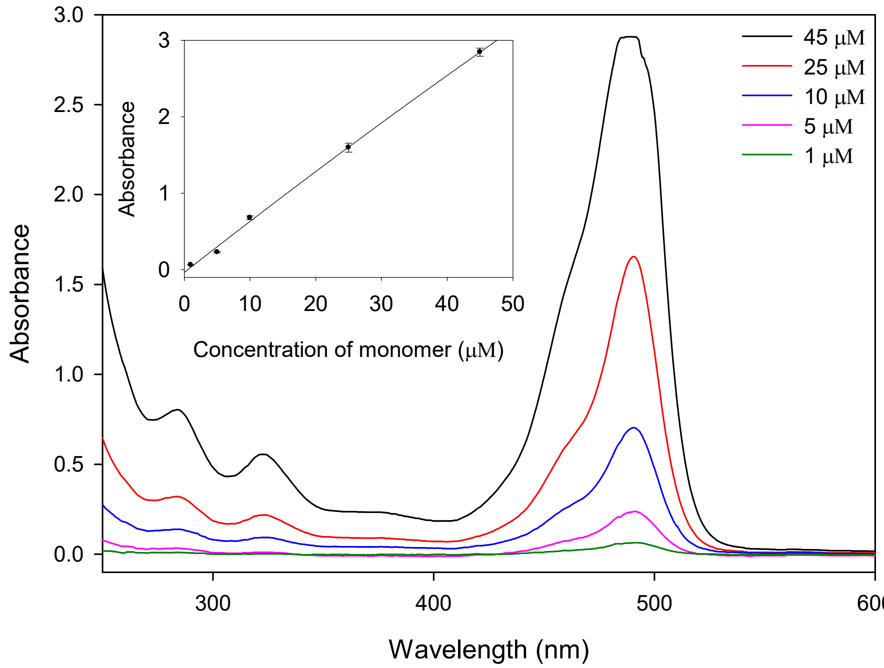

Figure S3: UV-Vis spectra of F-NVOC-PEG$_{3400}$-methacrylamide (45, 25, 10, 5 and 1 μM) in PBS where inset shows average absorbance between ± 5 nm of peak wavelength of 492 nm *versus* the monomer concentration (molar extinction coefficient at 492 nm = 63,900 ± 00 $M^{-1}cm^{-1}$)

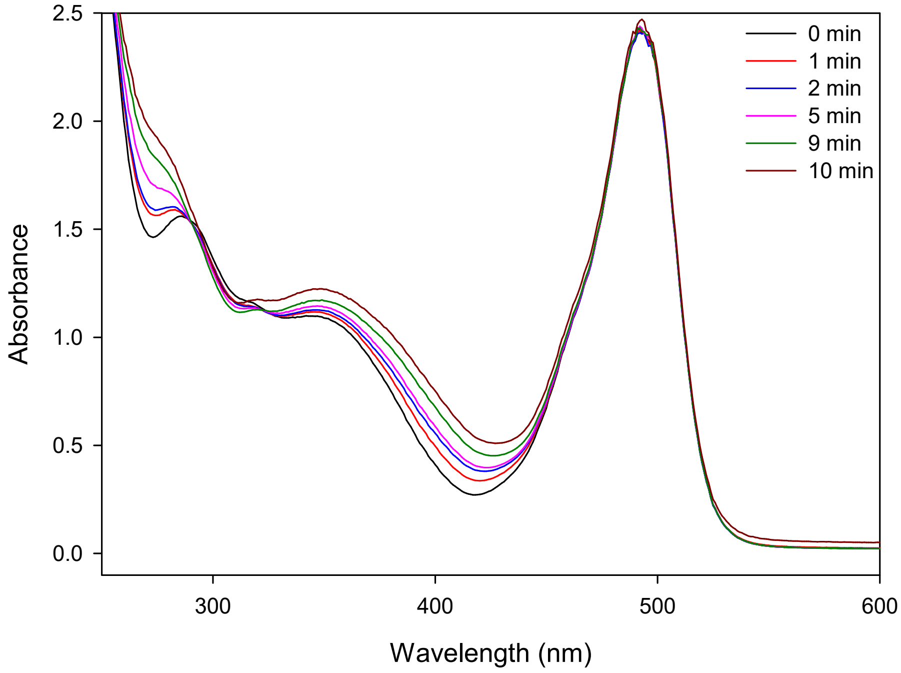

Figure S4: UV-Vis spectra of 40 μM F-NVOC-PEG$_{3400}$-methacrylamide monomer in PBS with increasing irradiation time

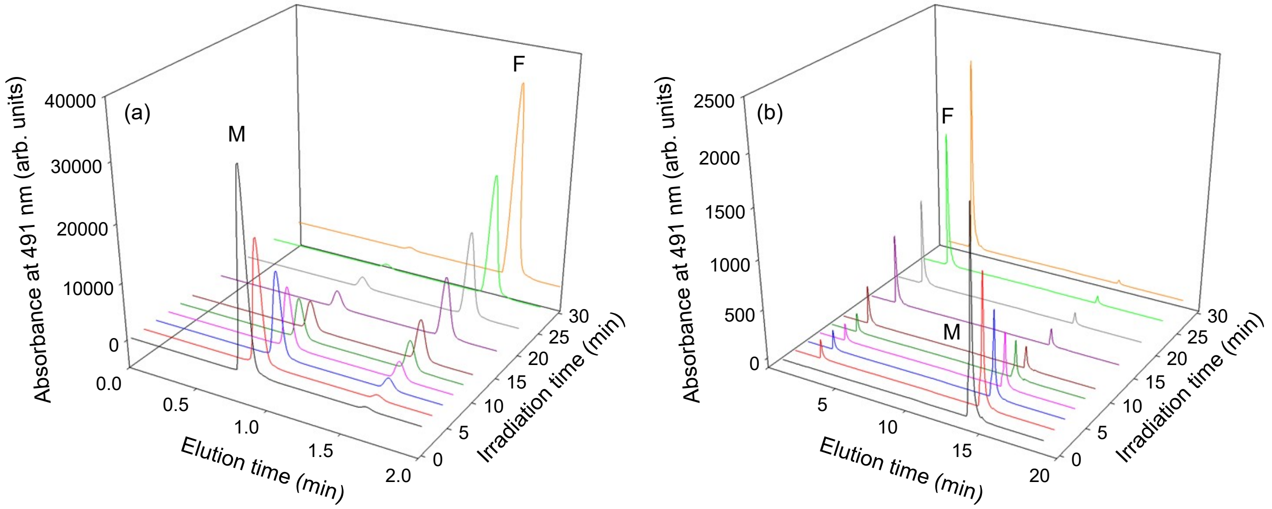

Figure S5: HPLC waterfall plots of 1 mg/ml (a) F- NVOC-allylamide and (b) F-NVOC-PEG$_{400}$-methacrylamide dissolved in acetonitrile showing increase in fluorescein (F) and decrease in photolabile monomer (M) concentrations for 0 to 30 min of irradiation time

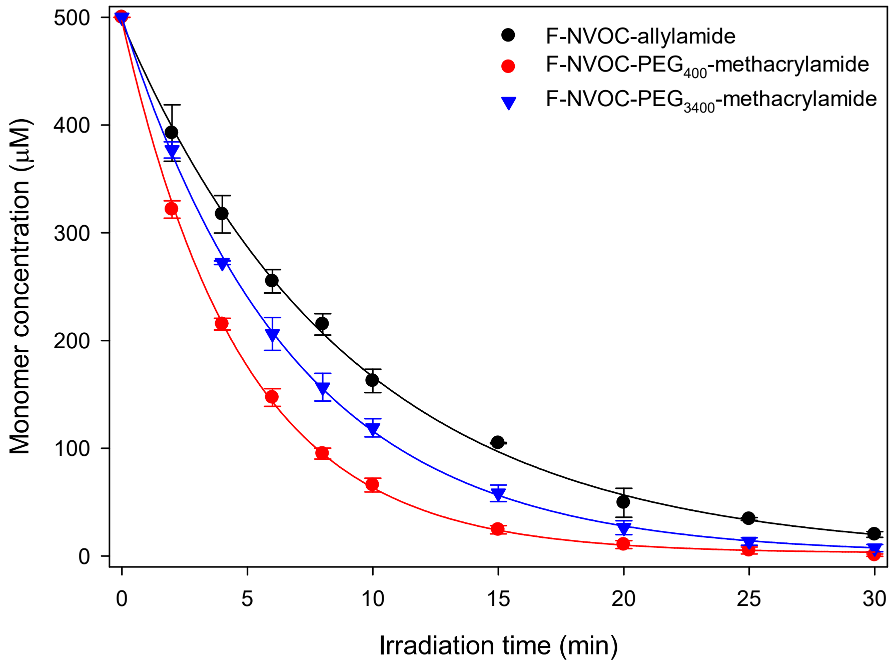

Figure S6: Plot showing monomer concentration, determined by the peak area corresponding to M in chromatograms and molar extinction coefficient of monomer solutions, as a function of irradiation time

Table S1: Summary of percentage of the photolabile monomer incorporated in hydrogel films (where the total concentration of monomers in precursor solutions was 10% w:v)

Sample number	Monomer	Molar % in precursor solution	Estimated molar % in hydrogel	% Monomer incorporation		
				Values	Average	Standard deviation
**1, 10**	F-NVOC	5	1.45, 1.35	29, 27	28	1.4
**2, 11**		10	3.1, 3.3	31, 33	32	1.4
**3, 12**		15	6.75, 4.35	45, 29	37	11.3
**4, 13**	F-NVOC-PEG_{400}-methacrylamide	5	3.55, 3.5	71, 70	70.5	0.7
**5, 14**		10	8.2, 7.2	82, 72	77	7.1
**6, 15**		15	9.9, 9.15	66, 61	63.5	3.5
**7, 16**	F-NVOC-PEG_{3400}-methacrylamide	5	3.35, 2.95	67, 59	63	5.7
**8, 17, 19-21**		10	7.1, 6.3, 5.9, 6.2, 7.0	71, 63, 59,62,70	65	5.2
**9, 18**		15	8.25, 9.9	55, 66	60.5	7.8

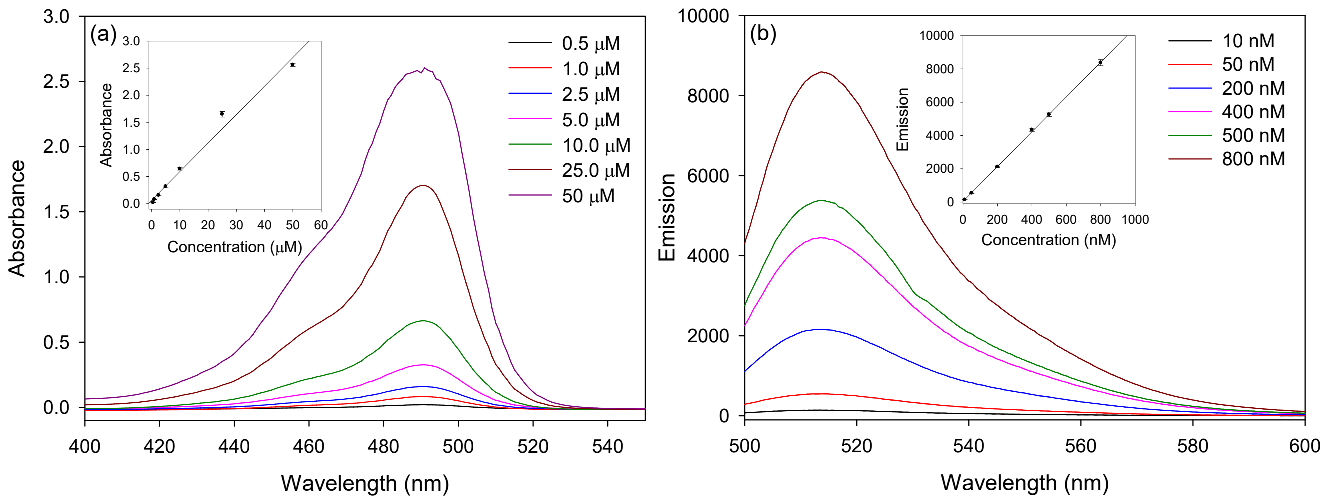

Figure S7: (a) UV-Vis and (b) fluorescence emission spectra (excitation wavelength of 470 nm) of fluorescein in PBS where insets show peak absorbance (averaged over 485-495 nm, molar extinction coefficient at 490 nm = 52,600 ± 3,200 $M^{-1}cm^{-1}$) and emission (averaged over 509-519 nm, molar emission coefficient at 514 nm = (10.4 ± 0.1) × 10^9 $M^{-1}cm^{-1}$) *versus* fluorescein concentration

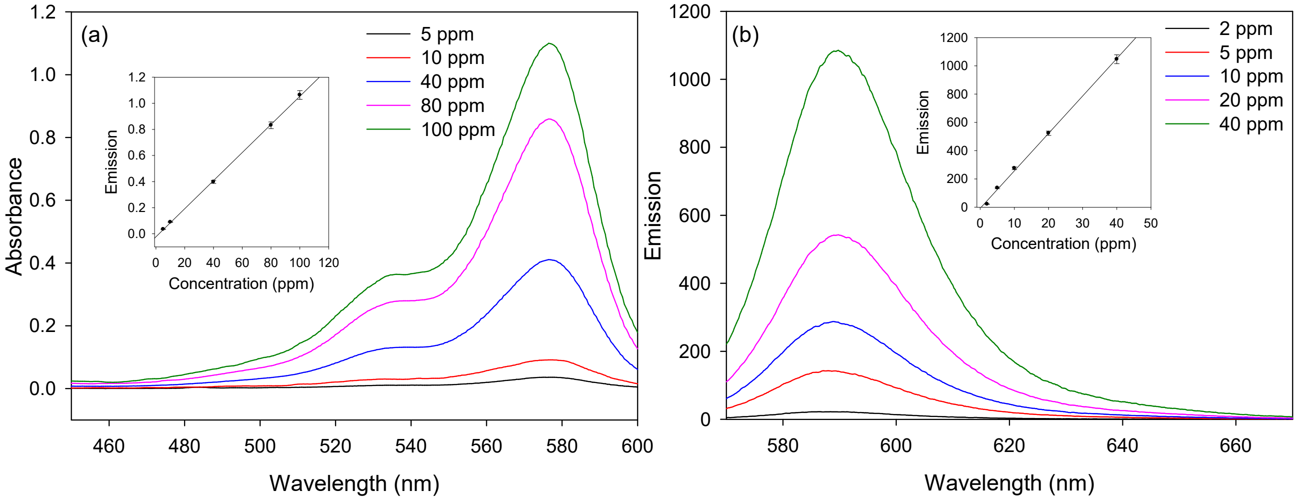

Figure S8: (a) UV-Vis and (b) fluorescence emission spectra (excitation wavelength of 540 nm) of rhodamine-streptavidin in PBS where insets show peak absorbance (averaged over 572-582 nm, molar extinction coefficient at 577 nm = 650,602 ± 6,024 $M^{-1}cm^{-1}$) and emission (averaged over 585-595 nm, molar emission coefficient at 590 nm = (1.6 ± 0.04) × 10^9 $M^{-1}cm^{-1}$) *versus* rhodamine-streptavidin concentration
